# The regulatory relationship between transcription factor STAT3 and noncoding RNA

**DOI:** 10.1186/s11658-023-00521-1

**Published:** 2024-01-03

**Authors:** Siyi Liu, Wentao Li, Lin Liang, Yanhong Zhou, Yanling Li

**Affiliations:** 1 Department of Nuclear Medicine, Hunan Cancer Hospital and The Affiliated Cancer Hospital of Xiangya School of Medicine, Central South University, Changsha, 410013 China; 2 Cancer Research Institute, Basic School of Medicine, Central South University, Changsha, 410011 Hunan China

**Keywords:** circRNA, LncRNA, microRNA, STAT3, Transcription factor

## Abstract

Signal transducer and activator of transcription 3 (STAT3), as a key node in numerous carcinogenic signaling pathways, is activated in various tumor tissues and plays important roles in tumor formation, metastasis, and drug resistance. STAT3 is considered a potential subtarget for tumor therapy. Noncoding RNA (ncRNA) is a special type of RNA transcript. Transforming from “junk” transcripts into key molecules involved in cell apoptosis, growth, and functional regulation, ncRNA has been proven to be closely related to various epithelial–mesenchymal transition and drug resistance processes in tumor cells over the past few decades. Research on the relationship between transcription factor STAT3 and ncRNAs has attracted increased attention. To date, existing reviews have mainly focused on the regulation by ncRNAs on the transcription factor STAT3; there has been no review of the regulation by STAT3 on ncRNAs. However, understanding the regulation of ncRNAs by STAT3 and its mechanism is important to comprehensively understand the mutual regulatory relationship between STAT3 and ncRNAs. Therefore, in this review, we summarize the regulation by transcription factor STAT3 on long noncoding RNA, microRNA, and circular RNA and its possible mechanisms. In addition, we provide an update on research progress on the regulation of STAT3 by ncRNAs. This will provide a new perspective to comprehensively understand the regulatory relationship between transcription factor STAT3 and ncRNAs, as well as targeting STAT3 or ncRNAs to treat diseases such as tumors.

## Introduction

Signal transducer and activator of transcription 3 (STAT3) is one of the seven members of the STAT family, being involved in regulating cell growth, differentiation, and survival [1, 2]. As the core regulator of the antitumor immune response, STAT3 can promote the production of immunosuppressive factors and participates in various biological processes, such as cell proliferation, differentiation, angiogenesis, and immune suppression [3, 4]. About 75% of the human genome is transcribed into RNA, but only 3% is transcribed into protein-encoding mRNA, while most will be transcribed into noncoding RNA (ncRNA) [5, 6]. At present, the most well-studied “classical ncRNA” types mainly include long noncoding RNA (lncRNA), microRNA (miRNA), and circular RNA (circRNA). They are widely recognized as various cancer biomarkers [7–9]. miRNAs can bind to mRNA to inhibit translation, and it is estimated that a single miRNA might simultaneously regulate hundreds of mRNA sequences [10]. Ongoing research has identified lncRNAs as the main regulatory factors for transcription and translation [11, 12], and their roles and mechanisms in various cancers have also been widely studied. circRNAs are circular RNAs produced by back-splicing of introns, exons, or intergenic regions [13]. The structural stability of circRNA also plays an important role in transcriptional regulation, intercellular information transmission, and translation processes [14–16].

Previous studies have mainly focused on the regulatory effect and mechanism of ncRNAs on the transcription factor STAT3. However, in recent years, researchers have shifted their attention to the regulation by STAT3 on ncRNAs and its mechanism. With the continuous recognition of the molecular mechanisms underlying the regulation of ncRNAs by STAT3 and its impact on tumor occurrence and development, new targeted anticancer strategies based on STAT3 or ncRNAs are gradually being developed, which might also provide potential therapeutic targets for cancer [17]. Therefore, it is important to summarize the research progress on the regulation by STAT3 on ncRNA and its mechanism to comprehensively understand STAT3’s function. In this review, we summarize the regulatory effects and possible mechanisms of STAT3 on lncRNAs, miRNAs, and circRNAs. At the same time, we provide an update on the progress of research into ncRNA-mediated regulation of STAT3, providing a basis for a comprehensive understanding of the mutual regulatory relationship between transcription factor STAT3 and ncRNAs, especially the mechanism by which STAT3 regulates ncRNAs.

## Transcription factor STAT3 directly binds to the LncRNA promoter to upregulate lncRNA expression, thereby affecting the biological phenotype and disease progression of cells

Studies have shown that transcription factor STAT3 can upregulate the expression of lncRNAs by directly binding to their promoters. LncRNAs, in turn, upregulate the expression of target genes by sponging miRNAs, ultimately promoting the proliferation, migration, and invasion of tumor cells [17–20], or regulating the differentiation of Th17 and M2 macrophages [21, 22], promoting the occurrence and development of immune-related diseases.

### STAT3 directly binds to lncRNA promoters to upregulate lncRNA expression, which sponge miRNAs to affect downstream target genes and regulate the biological phenotype of tumor cells

STAT3 is a transcription factor that is overactivated in most human cancers. The activation of signaling pathways such as JAK/STAT3 and vascular endothelial growth factor receptor 2 (VEGFR2)/STAT3/PD-L1 can promote STAT3 phosphorylation. JAKs can be activated by various receptors, including interleukin-6 (IL-6), IL-6 family cytokines, Toll-like receptors (TLRs), and G protein coupled receptors (GPCRs) [23]. Activated JAKs, such as JAK1 and JAK2, phosphorylate STAT3 at the Tyr705 site, leading to recruitment and activation of STAT3 in the cytoplasm and translocation to the nucleus. Meanwhile, serine/threonine kinases mediate Ser727 phosphorylation of STAT3, enhancing its transcriptional activity [24]. It has been reported that STAT3 can promote the progression of breast cancer (BC), non-small cell lung cancer (NSCLC), colorectal cancer (CRC), and other cancers after phosphorylation.[25–28]. Recently, studies reported a close correlation between STAT3 and abnormal upregulation of specific lncRNAs in these cancers. Some lncRNAs can serve as competitive endogenous RNAs (ceRNAs) that function as miRNA molecular sponges [29]. They inhibit binding of miRNAs to their downstream cancer-related target genes by sponging the miRNAs, thereby upregulating the expression of the target genes. An et al. suggested that STAT3 can directly bind to the P2 site in the lncRNA *LINC006688* promoter region, inducing upregulation of *LINC00668* expression. Subsequently, *LINC00668* upregulated *KLF7* (encoding KLF transcription factor 7) expression by sponging miR-193a, thereby promoting NSCLC cell proliferation, migration, and invasion, and inhibiting cell apoptosis [18]. Wang and colleagues found that, in human BC cell lines, STAT3 binds to the promoter of lncRNA *TINCR*, thereby enhancing its transcription. Subsequently, *TINCR* upregulates the expression of the miR-503-5p target gene, *EGFR* (encoding epidermal growth factor receptor), by adsorbing miR-503-5p, ultimately promoting tumor development [17]. In addition, Zheng and colleagues confirmed that lncRNA *RHPN1-AS1* is overexpressed in CRC cell lines. STAT3 promotes the transcription of *RHPN1-AS1* by binding to the upstream transcription start site (TSS) at −1915 to −1905 in the *RHPN1-AS1* promoter region. Subsequently, *RHPN1-AS1* accelerates the progression of CRC through sponging miR-7-5p-mediated *O*-GlcNAcy transfer (OGT) [19]. In summary, JAK1 or JAK2 is activated by various receptors such as IL-6, IL-11, LIF, GPCR, TLRs, etc., in most cancer cells. After activation, JAKs mediate the activation of STAT3 by promoting its phosphorylation, upregulating its expression [24]. Subsequently, STAT3 can promote transcription of lncRNAs and upregulate their expression by directly binding to their promoters. Ultimately, the lncRNAs induce tumor cell proliferation, migration, and invasion by adsorbing miRNAs to upregulate the expression of downstream target genes.

To date, research on the regulation of lncRNA expression by STAT3 has mainly focused on the upregulation of lncRNA expression by STAT3 binding to its promoter. However, further research is needed to determine whether STAT3 can enhance or inhibit the expression of LncRNAs via other regulatory mechanisms, thereby affecting the biological phenotype of tumor cells.

### STAT3 directly binds to an lncRNA promoter to upregulate the expression of lncRNA, thus forming a STAT3/lncRNA/miRNA positive feedback loop, thus affecting the biological phenotype of tumor cells

**Fig. 1:**
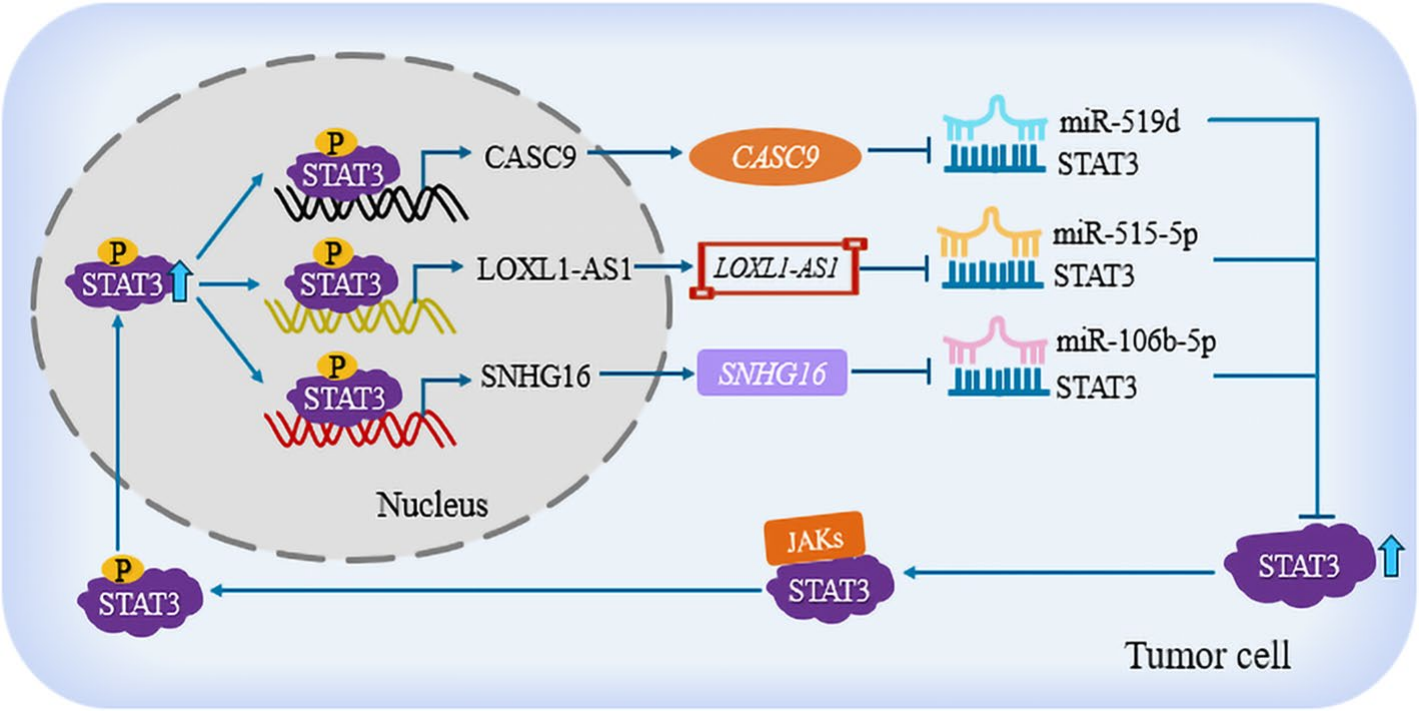
STAT3 binds to lncRNA promoters to upregulate their expression, forming a STAT3/lncRNA/miRNA positive feedback loop. In tumor cells, STAT3 upregulates lncRNA expression, such as *CASC9* and *LOXL1-AS1*, and SNHG16, by binding to their promoter. Subsequently, the lncRNAs relieve the inhibition of miRNAs (such as miR-519d, miR-515-5p, and miR-106b-5p) on STAT3 by adsorbing them, thereby restoring the expression of STAT3, forming a STAT3/lncRNA/miRNA positive feedback loop, and promoting the occurrence and development of tumors

LncRNAs have been revealed to play important roles in a wide range of fields, such as human tumors and cardiovascular system disorders [30], and the transcription of lncRNAs might be regulated by certain transcription factors [18], such as STAT3. Liu et al. confirmed that STAT3 and lncRNA *CASC9* are highly expressed in glioma specimens and cells, and STAT3 can directly bind to the second site (P2) of the *CASC9* promoter to promote its expression. At the same time, *CASC9* upregulates the expression of STAT3 through sponging miR-519d, forming a positive feedback loop of STAT3/CASC9/miR-519d. Ultimately, it enhances tumor proliferation, invasion, and migration in vitro, and promotes tumor growth in vivo [31]. Yang et al. showed that STAT3 is an upstream factor for lncRNA *SNHG16* transcriptional activation in vascular smooth muscle cells (VSMCs). STAT3 promotes *SNHG16* expression by binding to the E1 site of the promoter. Meanwhile, lncRNA *SNHG16* upregulates the expression of STAT3 by inhibiting the binding of miR-106b-5p to STAT3, ultimately forming a complex deteriorating loop in abdominal aortic aneurysm (AAA) by regulating VSMCs [20]. Meanwhile, Xie et al. found that STAT3 transcriptionally activates lncRNA *LOXL1-AS1*, upregulating its expression in VSMCs and human umbilical vein endothelial cells (HUVECs) by binding to the promoter. In addition, *LOXL1-AS1* sponges miR-515-5p to inhibit its binding with STAT3, thereby increasing the expression of STAT3, forming a *LOXL1-AS1*/miR-515-5p/STAT3 positive feedback loop, and promoting cell proliferation and migration in atherosclerosis (AS) [32]. (Fig. 1). In summary, STAT3 can directly upregulate its expression by binding to the promoters of lncRNAs. Subsequently, the lncRNAs absorb miRNAs targeting STAT3, thereby increasing the expression of STAT3, and forming a STAT3/lncRNA/miRNA positive feedback loop, which promotes the occurrence and development of tumors.

These findings strongly support STAT3 as a downstream target for lncRNAs and as a transcription factor involved in the upregulation of lncRNAs, forming a feedback loop. At the same time, the proposed loop might also provide reference significance for the study of STAT3 and other gene regulatory mechanisms, and provide valuable treatment strategies for cancer. However, it is unclear whether there are other downstream miRNA target genes that are involved in the development of gliomas, AAA, and AS, which also use feedback loops to ultimately regulate the occurrence and development of tumors. These questions should be addressed in future studies.

### STAT3 directly binds to lncRNA promoters to upregulate their expression, which promotes cell differentiation by regulating the expression of some Th17 or M2 macrophage differentiation-related genes, thereby promoting the occurrence and development of immune-related diseases

**Fig. 2:**
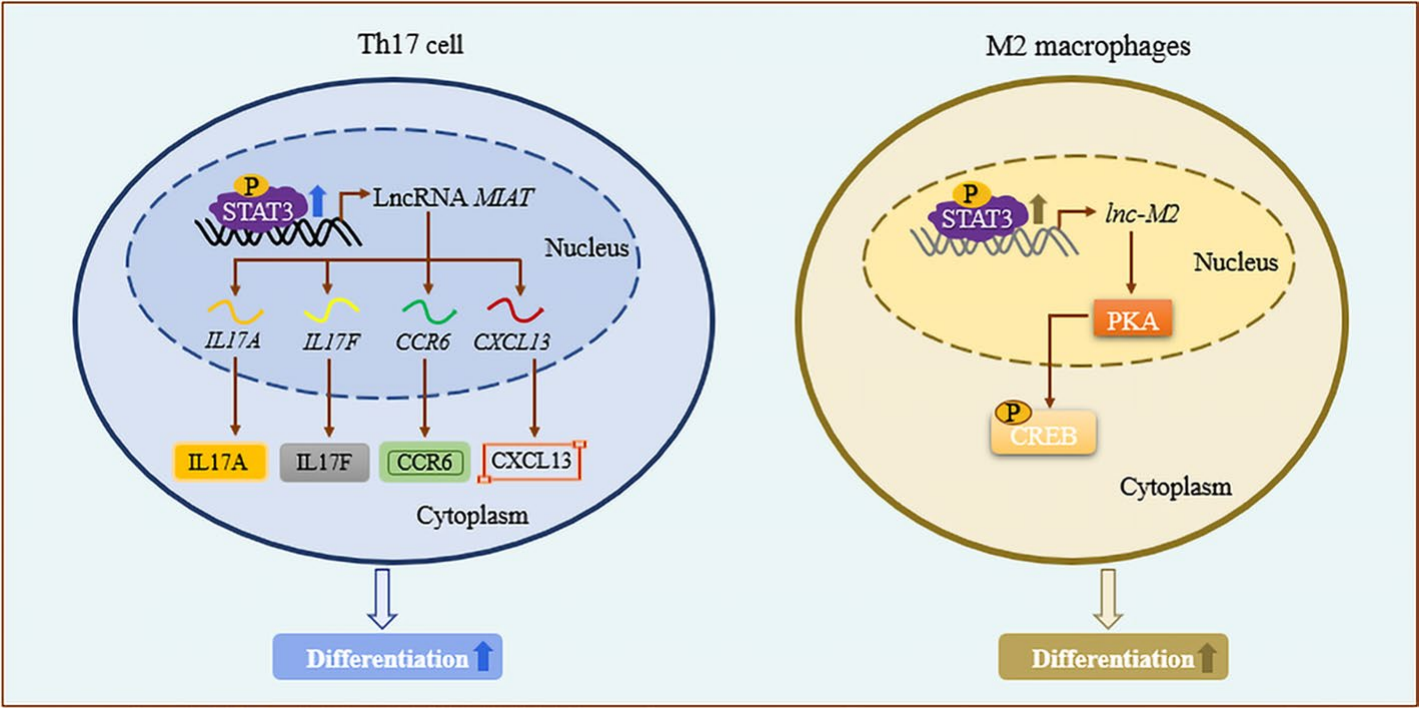
STAT3 binds to lncRNA promoters to upregulate their expression, promoting Th17 or M2 macrophage differentiation. In Th17 and M2 macrophages, STAT3 directly binds to the promoters of lncRNAs *MIAT* and *lnc-M2* to upregulate their expression. The lncRNAs promote the differentiation of Th17 and M2 macrophages by upregulating the expression of differentiation-related genes

STAT3 is a key regulatory factor for human Th17 cell differentiation, and abnormalities of its signaling pathway are key factors in chronic inflammation and autoimmune diseases [33, 34]. After STAT3 binds directly to the promoter of an lncRNA and upregulates its expression, the lncRNA can promote cell differentiation by regulating the expression of genes or proteins related to Th17 and M2 macrophage differentiation, ultimately promoting the occurrence and development of immune-related diseases [21, 22]. Khan et al. found that STAT3 directly binds to −1500 to +250 from *MIAT* TSS, which induces its expression in the early stages of Th17 cell differentiation. Upregulated *MIAT* resides in the nucleus and regulates the expression level of protein kinase C α (PKC-α). PKC-α is one of the early signaling molecules activated in response to T cell activation. It regulates the expression of several key Th17 genes, including *IL17A* (encoding interleukin 17A), *IL17F* (encoding interleukin 17F), *CCR6* (encoding C–C motif chemokine receptor 6), and *CXCL13* (encoding C-X-C motif chemokine ligand 13), through SMAD regulation of TGF signal transduction [35], ultimately regulating human Th17 differentiation and mediating the occurrence of autoimmune diseases such as rheumatoid arthritis (RA) and psoriasis [21]. In addition, Chen et al. showed that *lnc-M2* is highly expressed in M2 macrophages. STAT3 promotes the transcription of *lnc-M2* and upregulates the epigenetic histone modification marker at the promoter of *lnc-M2*, which suggested that STAT3 activates *lnc-M2* and promotes the differentiation of M2 macrophages through the protein kinase A (PKA)/cAMP response element binding protein (CREB) pathway, ultimately mediating the core role of macrophages in the pathogenesis of asthma and allergy, tumorigenesis, and atherosclerosis [22] (Fig. 2). The above studies confirmed that STAT3 directly binds to the promoters of lncRNAs *MIAT* and *lnc-M2*. Upregulated expression of these lncRNAs promotes the differentiation of Th17 and M2 macrophages by upregulating the expression of differentiation-related genes, ultimately accelerating the development of autoimmune diseases such as RA and asthma.

Determination of the specific molecular mechanism of STAT3 in the differentiation process of Th17 and M2 macrophages could increase our understanding of the role of STAT3. Meanwhile, it also provides us with new research ideas: Can STAT3 regulate the differentiation of other tumor-related immune cells? Does it have a more profound impact on the tumor microenvironment? Currently, further work is needed to clarify how STAT3 upregulates lncRNA expression and affects the differentiation of Th17 and other immune cells, mediating the development of autoimmune diseases. This might provide potential therapeutic methods to alleviate autoimmune diseases.

## STAT3 affects miRNA expression by binding to miRNA promoters, promoting the occurrence and development of diseases

Current research indicates that STAT3 not only has the ability to directly regulate lncRNAs, but also exerts a regulatory role on miRNAs. STAT3 can upregulate the expression of miRNAs by directly binding to their promoters, thereby forming a STAT3/miRNA/IL-6 loop or affecting the expression of downstream target genes, ultimately promoting tumor cell proliferation, invasion, and migration, and accelerating tumor growth [36–39]. In addition, specific miRNAs have conserved STAT3 binding sites, which prevent STAT3 from binding to them, leading to downregulation of miRNA expression and ultimately promoting tumor cell invasion and migration [40, 41].

### STAT3 directly combines with miRNA promoters to upregulate miRNA expression, thus forming STAT3/miRNA/IL-6 positive feedback loops or affecting downstream target genes, thus affecting the occurrence and development of tumors and diseases

STAT3 enhances the expression of miRNAs by directly binding to their promoters. After the miRNA is upregulated, it can induce the production of IL-6 to form a STAT3/miRNA/IL-6 loop [36, 37], or can regulate the expression of downstream target genes, thereby promoting tumor cell growth and accelerating cancer progression [38, 39].

#### STAT3 directly binds to the promoters miR-223 and miR-29a-5p to upregulate their expression, thus forming STAT3/miR-223/IL-6 or STAT3/miR-29a-5p positive feedback loops, and regulating the biological phenotype of tumor cells

**Fig. 3:**
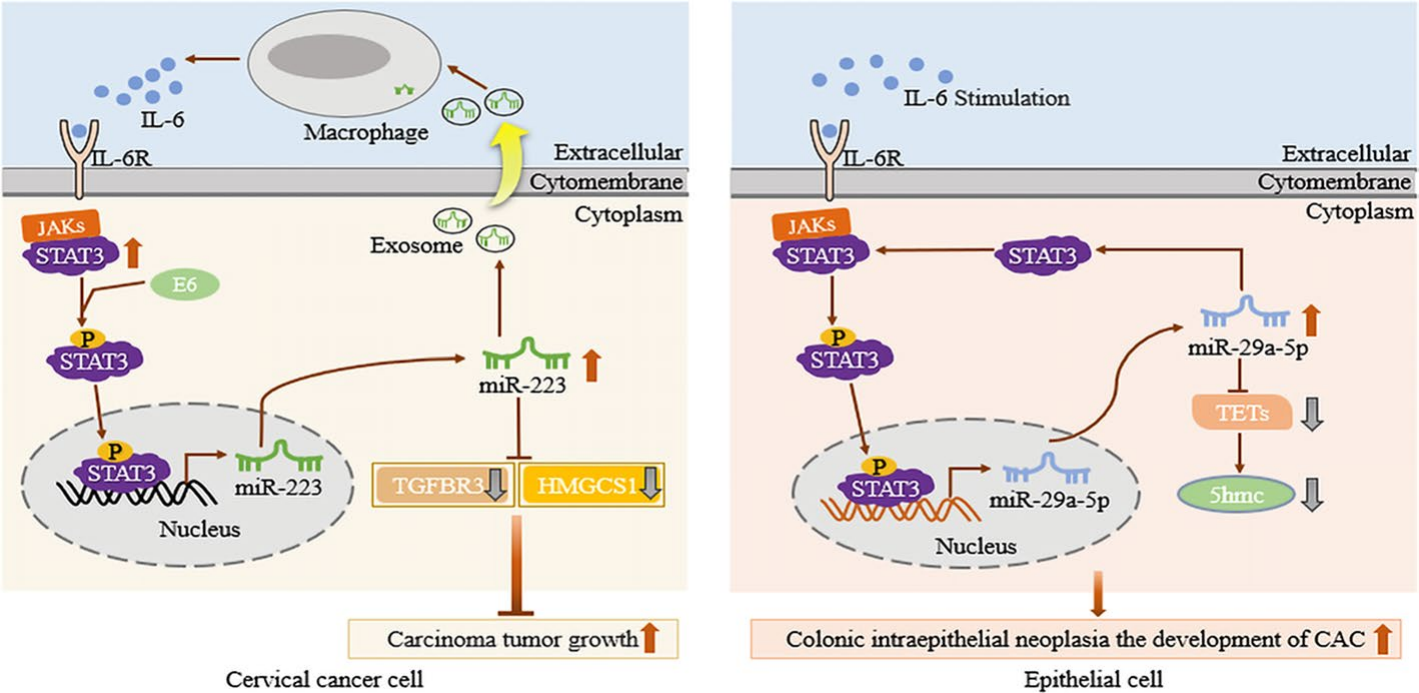
STAT3 directly binds to miRNA promoters to upregulate their expression, forming STAT3/miR-223/IL-6 or STAT3/miR-29a-5p loops. In CSSC and CAC, STAT3 upregulates miRNA expression by binding to the miR-223 and miR-29a-5p promoters. Subsequently, exosomal miR-223 induces monocytes/macrophages to secrete IL-6, which in turn mediates the upregulation of STAT3 expression in CSSC cells. Meanwhile, overexpression of miR-29a-5p also increases the expression of STAT3. Finally, a positive feedback loop is formed to promote the growth of CSCC and CAC tumors

STAT3/miRNA/IL-6 feedback loops promote tumor cell proliferation and epithelial–mesenchymal transition (EMT), and accelerate the development of various diseases, such as cervical squamous cell carcinoma (CSCC) [36]. Zhang and colleagues found that the expression of STAT3 was significantly increased in CSCC. STAT3 binds to the *MIR233* promoter to upregulate miR-223 expression. Subsequently, miR-223 targets the 3′-untranslated regions (UTRs) of *TGFBR3* (encoding transforming growth factor beta receptor 3) and *HMGCS1* (encoding 3-hydroxy-3-methylglutaryl-CoA synthase 1), significantly downregulating their expression, and ultimately promoting CSCC tumor growth in vitro and in vivo. In addition, exosomal miR-223 from CSCC cells induces monocytes and macrophages to secrete IL-6 in a co-culture system in vitro, which in turn mediated enhance STAT3 activity in CSCC cells, thus forming a positive feedback loop to accelerate CSCC deterioration [36]. Wang et al. also confirmed that, in colorectal cancer (CAC) cell lines, activation of STAT3 by the inflammatory factor IL-6 might lead to upregulation of miR-29a-5p in epithelial and immune cells, as well as inhibition of the expression of the miR-29a-5p target gene *TET3* (encoding Tet methylcytosine dioxygenase 3), thereby inducing a decrease in 5-hydroxymethylcytosine (5hmC) in epithelial cells. In addition, overexpression of miR-29a-5p can also increase the expression of STAT3, forming a positive feedback loop of STAT3/miR-29a-5p, leading to a reduction of TET3 and 5hmC levels, and promoting the development of CAC [37] (Fig. 3). The above research has clarified the role of STAT3/miR-223/IL-6 and STAT3/miR-29a-5-p feedback loops in the occurrence and development of CSCC and CAC, and marking them as potential therapeutic targets in CSCC and CAC. In addition, clarification of the feedback loop also provides a new perspective on the regulatory relationship between STAT3 and miRNAs.

However, these studies also have limitations. Current research has confirmed that there is a positive feedback loop between STAT3 and miR-29a-5-p; however, the specific regulatory mechanism between them has not been determined, thus requiring further exploration. Meanwhile, the above studies were conducted in vitro, and it is necessary to supplement relevant in vivo animal model experiments.

#### STAT3 directly binds to miRNA promoters to upregulate their expression, thereby affecting downstream target genes and promoting the occurrence and development of diseases

Research has shown that STAT3 inhibits the expression of downstream genes by upregulating miRNA expression, which contributes to the occurrence and development of diseases, such as hepatocellular carcinoma (HCC) and ischemic retina [38, 39]. Wang et al. found that, in mouse liver tumors and primary human HCC, IL-6 activates STAT3 and upregulates its expression. Subsequently, STAT3 directly binds to the promoter region of *MIR23A* and enhances miR-23a expression. miR-23a then directly targets and reduces the expression of *PGC1A* (encoding PPARG coactivator 1 alpha) and *G6PC* (encoding glucose-6-phosphatase), leading to a decrease in glucose production, which might ultimately contribute to the survival of tumor cells under hypoxic conditions [38]. A study by Gutsaeva et al. confirmed that the expression of miR-21 in human retinal endothelial cells (HRECs) exposed to hypoxia is dependent on STAT3. STAT3 can upregulate *MIR21* expression by directly binding to its transcription start, thus promoting the activation of the STAT3/miR-21 signaling pathway. Upregulated miR-21 then inhibits *TIMP3* (encoding tissue inhibitor of matrix metalloproteinase-3) expression, thereby promoting the occurrence of retinal neovascularization, and possibly causing ischemic retinopathy [39]. In summary, STAT3 can directly bind to the promoter regions of *MIR23A* and *MIR21* to upregulate their expression. miR-23a and miR-21 in turn inhibit the expression of *PGC1A*, *G6PC*, and *TIMP3*, thereby promoting tumor growth or accelerating the development of ischemic retinopathy.

The above research confirmed that STAT3 regulates gluconeogenesis and retinal neovascularization in HCC and retinal diseases, respectively, by upregulating specific miRNAs, providing strong support for the diversity of STAT3 functions. Studies have also found that STAT3 mediates changes in the expression level of specific miRNAs in different tumor cells, leading to different results. For example, miR-21 has a dual role in angiogenesis, not only promoting the occurrence of retinal neovascularization, but also inhibiting the generation of choroidal neovascularization [42]. The reason for this difference might be the different properties of retinal and choroidal endothelial cells [39]; however, the specific molecular mechanism that leads to the difference requires further research.

### STAT3 can downregulate miRNA expression, forming a STAT3/miRNA/IL-6R positive feedback circuit, and regulating the biological phenotype of tumor cells

**Fig. 4:**
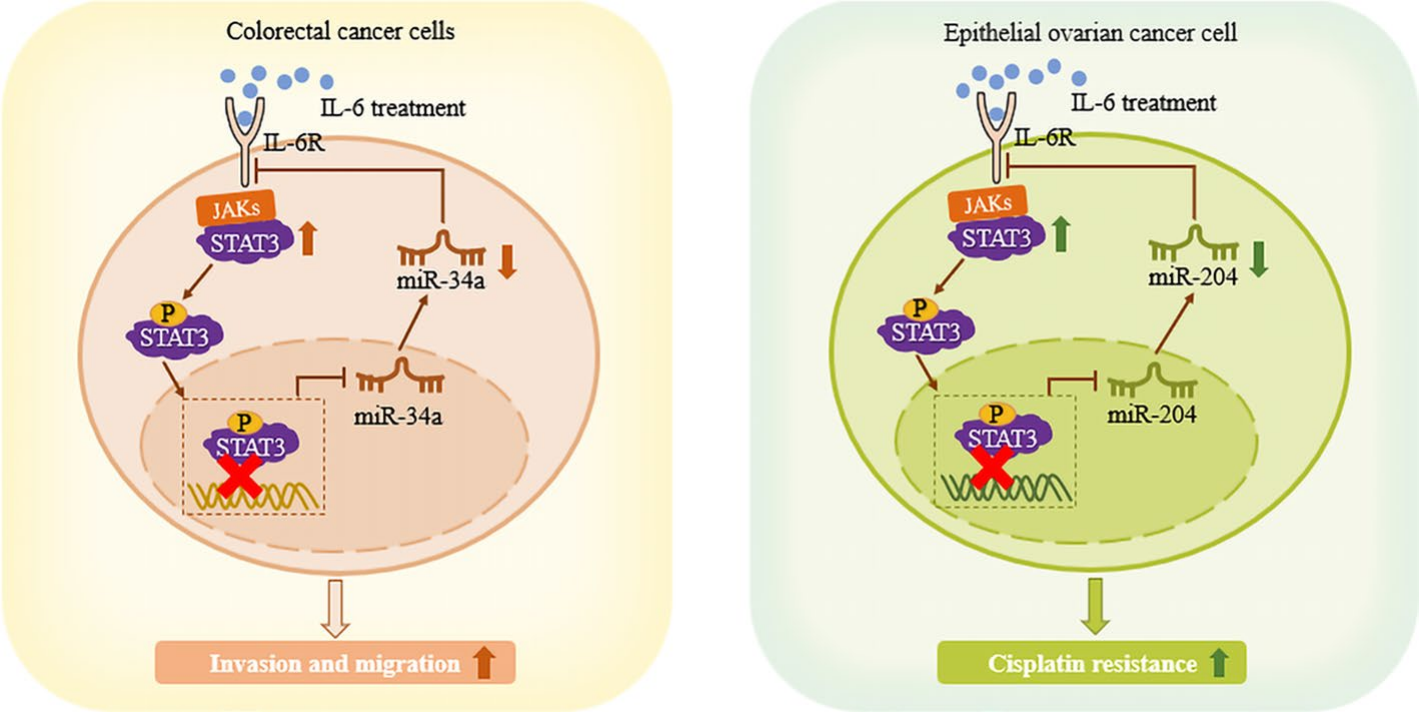
STAT3 can downregulate miRNA expression, forming a STAT3/miRNA/IL-6R positive feedback loop. STAT3 cannot bind to the miR-34a and miR-204 promoters, resulting in inhibition of miRNA expression. At the same time, after IL-6 stimulates CAC cells and EOC cells, the activity of IL-6R, as the direct target of miR-34a and miR-204, is enhanced, and then mediates IL-6 to upregulate the expression of STAT3, forming a positive feedback loop, and promoting the invasion and migration of colorectal cancer cells and the cisplatin resistance of EOC cells

There is ample evidence that STAT3 directly upregulates expression of miRNAs by binding to their promoters; however, a few studies have found that STAT3 can downregulate miRNA expression. A study by Rokavec et al. showed that exposure of human CRC cells to cytokine IL-6 activated STAT3, but the expression of miR-34a was inhibited. Examination of the *MIR34A* genome region showed a phylogenetically conserved STAT3 binding site in the first intron near the first exon, which prevented STAT3 from binding to the *MIR34A* promoter, resulting in direct inhibition of miR-34a expression. In addition, downregulation of miR-34a leads to less binding of miR-34a to its downstream target, *IL6R* (encoding IL-6 receptor), thereby upregulating IL-6R levels, which ultimately promoted IL-6-induced STAT3 expression, forming a STAT3/miR-34a/IL-6R feedback loop, and promoting tumor cell invasion and migration [40]. In addition, Zhu et al. found that exposure of epithelial ovarian cancer (EOC) cells to IL-6 activated STAT3 and upregulated its expression. Subsequently, STAT3 directly inhibited the expression of miR-204 through the conserved STAT3 binding site near the *TRPM3* (encoding transient receptor potential cation channel subfamily M member 3) promoter region upstream of miR-204. Inhibition of miR-204 enhanced the activity of IL-6R, as the direct target of miR-204, which prompted IL-6 to upregulate the expression of STAT3, forming a STAT3/miR-204/IL-6R positive feedback loop, ultimately promoting enhanced cisplatin resistance of EOC cells, inducing treatment failure and EOC recurrence [41] (Fig. 4).

Cisplatin is a commonly used chemotherapy drug for treating various types of cancer [43]. However, intrinsic or acquired resistance to cisplatin remains a major obstacle in the cancer treatment process [44]. A large number of studies currently indicate that STAT3 plays an important role in mediating drug resistance in cancer treatment [45]. Therefore, considering that the miR-34a/IL-6R/STAT3 pathway regulates cisplatin resistance in EOC cells proposed by the above study, we can also reflect on whether there are other miRNAs in EOC cells that can regulate STAT3 expression and thereby affect cisplatin resistance in EOC cells. This may be very beneficial for the treatment of EOC.

In summary, the presence of conserved STAT3 binding sites near the *MIR34A* and *MIR204* promoter regions means that their transcription is inhibited by STAT3, thereby enhancing the expression of IL-6R. IL-6R ultimately promotes the expression of STAT3, forming a STAT3/miRNA/IL-6R feedback loop, promoting tumor cell invasion and migration. The above research proves that the transcription factor STAT3 can not only promote miRNA expression but also inhibit miRNA expression. In addition, current research on the STAT3/miR-34a/IL-6R loop has not only clearly confirmed that it is necessary to maintain the mesenchymal phenotype of CRC cell lines but also detected its activation in BC and prostate cancer cell lines with mesenchymal characteristics [40]. This suggests that this loop might represent a new mechanism of carcinogenesis in cancer cells exhibiting a mesenchymal phenotype and might become a useful prognostic marker for cancer progression. However, it is unclear whether the STAT3/miR-34a/IL-6R loop plays the same role in different tumor cells, which requires further research.

## STAT3 directly binds to circRNA promoters to upregulate their expression, thereby forming a STAT3/circRNA positive feedback loop or adsorbing miRNA to affect downstream target genes and regulate the biological phenotype of tumor cells

STAT3 plays a significant regulatory role in the expression of various lncRNAs and miRNAs. However, there are far fewer cases of STAT3 regulating circRNA expression. Sun et al. showed that circ-LRIG3 is significantly upregulated in HCC. STAT3 can directly bind to the *circ-LRIG3* promoter, thereby increasing *circ-LRIG3* transcription activity. In turn, circ-LRIG3 forms a ternary complex with EZH2 (enhancer of zeste 2 polycomb repressive complex 2 subunit) and STAT3. After phosphorylation by AKT at serine residue 21 (pS21-EZH2), EZH2 can promote the methylation and phosphorylation of STAT3, thereby enhancing STAT3 activity, activating STAT3 signal transduction [46], and forming a positive feedback loop of STAT3/circ-LRIG3, which ultimately promotes the proliferation, migration, and invasion of HCC cells, and reduces cell apoptosis [47]. In addition, Wang and colleagues found that circCCDC66 is highly expressed in NSCLC cells [48]. STAT3 has been shown to bind to the *CCDC66* promoter site 1, activating *CCDC66* transcription into CCDC66 mRNA. And then CCDC66 mRNA is translated into CCDC66 protein, thus increasing the expression of CCDC66 protein and circCCDC66 in NSCLC cells [48]. Subsequently, circCCDC66, as a molecular sponge for miR-33a-5p, downregulates miR-33a-5p expression and inhibits its binding to the target gene *KPNA4* (encoding karyopherin subunit alpha 4). This upregulates *KPNA4* levels, ultimately promoting the proliferation, migration, and invasion of NSCLC cells, and inhibiting cell apoptosis [48]. STAT3 is considered an oncogenic gene or RNA transcription activator in various human cancers, such as cervical cancer, BC, and oral squamous cell carcinoma [49–51]. These studies confirmed the carcinogenic effect of STAT3 in NSCLC via circCCDC66 regulation. They also identified the molecular mechanism by which STAT3 binds to the *CCDC66* promoter at site #1, thereby promoting its expression in NSCLC cells. Subsequently, CCDC66 upregulated the expression of *KPNA4* by adsorbing miR-33a-5p, which might provide new ideas for molecular targeted therapy of NSCLC.

CircRNA, as an important noncoding RNA, also plays an important role in the development of cancer. However, there are currently few studies on the regulation of STAT3 expression by circRNA, and more research is needed to explore the regulatory role of STAT3 on circRNAs. In addition, the above research did not mention how STAT3 upregulates the expression of target genes via circRNAs, and how the target genes promote cancer development, which requires further research and exploration.

## LncRNAs regulate STAT3 expression directly or indirectly, affecting the biological phenotype of tumor cells or the occurrence and development of diseases

In HCC, BC, ovarian cancer (OV), and HCC, lncRNAs mainly alter the expression of STAT3 by directly binding to STAT3 [52–59], inhibiting the binding of miRNAs to downstream target genes [60–67], regulating the expression of IL-6, IL-11, and IL-23 [68–80], or regulating the protein level of STAT3 [81, 82], ultimately affecting the biological phenotype of tumor cells or the occurrence and development of diseases.

Firstly, LncRNAs can regulate STAT3 expression by directly binding to it. On the one hand, lncRNAs such as *FAL1*, *FEZF1-AS1*, *PVT1*, and *RP11-334E6.12* can upregulate STAT3 expression by binding to the STAT3 promoter and inducing STAT3 phosphorylation, thereby directly inducing tumor cell proliferation and inhibiting apoptosis [52–58, 83]. On the other hand, studies have confirmed that *lncRNA-p21* binding to STAT3 inhibits the phosphorylation of STAT3, thereby decreasing its stability, ultimately significantly inhibiting tumor cell proliferation and promoting apoptosis [59]. The above research determined that *lncRNA-p21* can downregulate the expression of STAT3 by binding to it, thereby inhibiting the proliferation ability of HNSCC cells. However, whether *lncRNA-p21* can act as a cancer promoting factor, like most lncRNAs, in the occurrence and development of HNSCC or other cancers, has not yet been determined, which requires further exploration.

Secondly, lncRNAs can regulate STAT3 expression by adsorbing miRNAs. Studies have shown that lncRNAs, such as *H19*, *HOST2*, *SNHG16*, *MIAT*, *NEAT1*, *GACAT3*, and *SNHG1*, serve as molecular sponges for miRNA, which can downregulate miRNA levels and inhibit their binding to STAT3 to enhance STAT3 expression, thereby accelerating the progression of cancers such as BC [60, 61], HCC [62–64], gastric cancer (GC) [65], renal cell carcinoma (RCC) [66], and CRC [67, 84], by inducing tumor cell proliferation, migration, and invasion. Meanwhile, studies have also found that lncRNAs such as *SNHG14*, *CASC2*, *LINC00982*, and *BCAR4* serve as molecular sponges for miRNAs, thereby inhibit the binding of miRNAs to downstream target genes, thus upregulating their expression, and indirectly regulating the expression of STAT3, ultimately affecting tumor cell proliferation and apoptosis [85–96]. However, most of these studies did not mention how the lncRNA/miRNA/STAT3 axis affects downstream signals and promotes tumor occurrence and development. And further exploration is still needed to provide relevant evidence in animal model experiments to verify the above conclusions.

Thirdly, lncRNAs can regulate STAT3 expression by regulating the lncRNA/ILs/STAT3 positive feedback loops or ILs/STAT3 signaling pathways. LncRNAs *TCF7* and *DANCR* can induce IL-6 expression, while *HEGBC* can upregulate IL-11 expression. LncRNAs activate STAT3 expression by inducing IL autocrine signaling. At the same time, STAT3 upregulates the expression of lncRNAs by binding to their promoters, forming lncRNA/IL-6/STAT3 or lncRNA/IL-11/STAT3 loops, which enhance the drug resistance of tumor cells [68, 97], or promoting tumor growth and metastasis in vivo and inhibiting tumor cell apoptosis [69, 70]. LncRNAs such as *LNRRIL6*, *TSLNC8*, *IL6ST-AS*, and *BHLHE40-AS1* can mediate changes in IL-6 expression. And *ATB* and *LINC01152* upregulate IL-11 and IL-23 expression, respectively, by binding to their promoters in HCC cells. Altered expression of these ILs regulates the expression of STAT3, ultimately affecting the proliferation, migration, and invasion of tumor cells [69, 71–80]. In summary, these research findings emphasize the important role of the lncRNA/IL/STAT3 axis in tumor development. Nevertheless, the above experiments were conducted in vitro on cancer cell lines. Future research might consider using lncRNAs in animal models for in vivo experiments to evaluate their safety and efficacy, and for further in-depth investigation.

Fourthly, lncRNAs can regulate the expression of STAT3 by regulating the expression of other downstream genes or proteins. Studies have shown that lncRNAs, such as *LOC645166* and *TRPM2-AS*, can upregulate the expression of STAT3 by regulating downstream genes or proteins other than miRNAs and ILs, such as GATA binding protein 3 (GATA3) and p38 mitogen-activated protein kinase, ultimately promoting tumor cell proliferation, migration, and invasion, or enhancing tumor tissue chemotherapy resistance [98–102]. In addition, other studies have confirmed that lncRNAs *UCA1*, *PRECSIT*, etc. can also regulate the expression of glutamate/aspartate transporter 1 (GLAST) or matrix metalloproteinase (MMP)-1, MMP-3, MMP-10, and MMP-13, which then downregulates the expression of STAT3, thereby inhibiting disease progression [103, 104]. However, in the process of finding new targets for cancer treatment, analyzing the relationship between lncRNA expression and cancer recurrence is also very important. Therefore, it would be meaningful to further explore the impact of lncRNAs regulating STAT3 expression on cancer recurrence in the future.

Finally, lncRNA alters STAT3 levels by stabilizing or downregulating STAT3 protein levels. Numerous studies have shown that specific lncRNAs, such as *UICC* and *NEAT1*, can protect STAT3 from degradation, thereby stabilizing STAT3 protein levels [81, 105]. *Lnc-UICC* can directly interact with phosphorylated STAT3 and improve the stability of STAT3 protein by protecting it from proteasome-dependent degradation [81]. In addition, STAT3 is a downstream molecule of lncRNA *NEAT1*, which can positively regulate STAT3 cell levels by reducing ubiquitination levels [105], ultimately promoting the growth and metastasis of cervical cancer tumors or accelerating RA progression. However, studies have also found that lncRNA *GAS5* can accelerate the degradation of STAT3 by promoting TNF receptor-associated factor 6 (TRAF6)-mediated ubiquitination. TRAF6 is a well-known ubiquitin ligase that has been reported to bind to STAT3 and mediate its ubiquitination [106]. The decrease in protein levels of STAT3 ultimately inhibits Th17 cell differentiation and inhibits the development of immune thrombocytopenia (ITP) in vivo [82]. The above research reflects the regulatory function of specific lncRNAs on inflammation, which suggests that further research on lncRNAs might reveal more potential functions. Meanwhile, most current research on lncRNA regulation of STAT3 expression has focused on lncRNAs altering the transcription or activation of STAT3, while there are relatively few studies on lncRNA regulation of STAT3 protein levels by promoting or inhibiting STAT3 degradation.

## MicroRNAs directly target STAT3 signaling pathway components, regulate STAT3 expression, and affect the biological phenotype of tumor cells

First, many studies have found that miRNAs can directly target STAT3, downregulate its expression, and inhibit the occurrence and development of tumors [107–110]. Therefore, the expression levels of some tumor suppressor miRNAs are typically significantly downregulated in tumor tissue samples. The significant downregulation of miR-361 and miR-4500 expression in extracapsular nodal spread (ECS) and acute lymphoblastic leukemia (ALL) cell lines leads to inhibition of their binding to the target gene STAT3, thereby enhancing STAT3 expression and ultimately accelerating the occurrence and development of ECS and ALL [107–109]. Second, miRNAs can target components of the STAT3 signaling pathway to alter STAT3 expression, ultimately regulating the biological phenotype of tumor cells [111–118]. miRNAs (e.g., miR-19a and miR-18a) can downregulate components of the STAT3 signaling pathway, such as suppressor of cytokine signaling 3 (SOCS3), SOCS5, and protein inhibitor of activated STAT3 (PIAS3), in tumor cells, thereby activating STAT3, and ultimately promoting tumor cell migration, invasion, and EMT [111–113]. However, when miR-9 and miR-218 downregulated the expression of IL-6 and Janus kinase 2 (JAK2), they inhibited the activation of STAT3, leading to the downregulation of STAT3 expression and thus inhibiting the growth of tumor cells [111, 114, 115].

As is well known, STAT3 is an important regulatory factor for cell proliferation and survival. However, in recent years, a large number of studies have shown that STAT3 also plays an important role in maintaining stem cells and their differentiation, thereby participating in the occurrence of diseases and various types of cellular carcinogenesis [119]. Cai et al. found that, in bone marrow-derived mesenchymal stem cells (BMSCs) co-cultured with cardiomyocytes, miR-124 inhibits the expression of STAT3 protein by targeting STAT3 mRNA. The downregulation of STAT3 levels will affect the expression of cardiac-specific markers such as atrial natriuretic peptide (ANP), troponin T (TNT), α-myosin heavy chain (α-MHC), and GATA-binding factor 4 (GATA-4), ultimately regulating the differentiation of BMSCs into cardiomyocytes. These findings will greatly improve the effectiveness of BMSC-based therapy for damaged myocardial repair and regeneration [120]. Zhang and colleagues confirmed that miR-7 was downregulated in breast cancer stem cells (BCSC) isolated from human breast cancer cell lines. miR-7 downregulates the expression of STAT3 by inhibiting the binding of SETDB1 to the STAT3 promoter, thereby inhibiting the expression of c-myc, twist, and miR-9. This leads to a decrease in the BCSC population, partial reversal of EMT in BC cells, and inhibition of invasion and metastasis of BC cells [121]. In addition, Jiang et al. also found that the expression of miR-1181 was significantly downregulated in cancer pancreatic tissue and cells. miR-1181 can reduce the transcriptional activity of STAT3 and SOX2 by targeting the 3′-UTR of them in cancer pancreatic cells, and inhibit STAT3 trans activators. This leads to downregulation of SOX2 and inhibition of the STAT3 pathway, ultimately inhibiting the CSC-like phenotypes in vitro and tumorigenicity in vivo [122].

The above research reveals the mechanism by which miRNAs regulate STAT3 expression and the subsequent impact of STAT3 on the differentiation characteristics of stem cells/tumor stem cells. In the future, these molecular targets may be used to treat diseases related to JAK-STAT3 signaling disorders or stem cells/CSCs differentiation. However, compared with the research on the mutual regulatory loop between lncRNAs and STAT3, there is currently sparse evidence to confirm the existence of feedback loops between specific miRNAs and STAT3. To further understand the close connection between miRNAs and STAT3 for the treatment of other diseases, further in-depth research is required.

## circRNAs, as miRNA sponges, can inhibit miRNA function, thereby promoting the expression of STAT3 and affecting the occurrence and development of diseases

Research has found that a large amount of circRNA can act as a miRNA sponge or RNA binding proteins (RBPs), thereby inhibiting miRNA function [123]. For example, circRNAs such as AKT3, RHOT1, SPARC, and UBE2Q2, as molecular sponges, can reduce the levels of miR-516b-5p, miR-106a-5p, etc., thereby hindering their binding to STAT3. The expression of STAT3 is ultimately upregulated, which plays a crucial role in promoting the occurrence and development of various cancers, as well as the proliferation, invasion, and migration of cancer cells [124–129]. These studies confirmed that certain specific circRNAs might be key tumor promoters and potential therapeutic targets for diseases [130]. Currently, most studies on the regulation of STAT3 by circRNAs have focused on upregulating the expression of STAT3 by adsorbing miRNAs. Recent studies have also found that, in squamous cell carcinoma (SCC), circFAT1 can prevent Src homology 2 domain-containing protein tyrosine phosphatase 1 (SHP-1) from dephosphorylating STAT3 and promotes STAT3 activation by binding to STAT3 in the cytoplasm [131]. However, compared with the mechanism by which lncRNAs regulate STAT3, there is relatively little research on the mechanism of circRNA regulation of STAT3. Is there any other molecular mechanism for circRNA to alter the expression of STAT3? If so, what are the specific mechanisms? These aspects require further exploration.

## Conclusions

STAT3 serves as a key signaling node for tumor cells, especially tumor infiltrating immune cells. At the same time, ncRNAs participate in various cell behaviors, and control cell apoptosis, cell growth, and cell function through the expression of regulator genes at the transcriptional, posttranscriptional, and epigenetic levels [5], which are related to tumorigenesis, drug resistance, and EMT of various types of cancer [88]. Therefore, understanding the regulatory relationship between STAT3 and ncRNAs and targeting STAT3 or ncRNAs is expected to provide valuable new strategies for cancer disease treatment and drug development. However, most studies have focused on ncRNA regulation of STAT3, and there is relatively little research on the molecular mechanism of STAT3 regulation of ncRNAs, especially circRNA expression. A deeper understanding of circRNA and the mechanisms regulating ncRNA expression may improve this situation. At the same time, the existing mechanistic research is more focused on in vitro experiments in relevant tumor cell lines. Therefore, it is necessary to use animal models in vivo to verify the results. Further in-depth research and analysis might provide potential directions and perspectives for cancer, disease diagnosis, and treatment (see Outstanding questions).

## Outstanding questions

- 1. Can the transcription factor STAT3 downregulate the expression of lncRNAs and thereby affect the biological phenotype of tumor cells? What is the specific molecular mechanism?
- 2. In addition to regulating the differentiation of Th17 and M2 macrophages, can STAT3 affect the differentiation of other tumor-related immune cells and have a more profound impact on the tumor microenvironment?
- 3. What is the specific role of the STAT3/miR-34a/IL-6R loop when activated in cancer cell lines with multiple mesenchymal phenotypes? Does this loop represent a new mechanism of carcinogenesis in these cancer cells?
- 4. In addition to circ-LRIG3 and circCCDC66, which other specific circRNAs are regulated by STAT3? Are there any other regulatory mechanisms?
- 5. In addition to the direct inhibition of miR-204 by STAT3 to mediate cisplatin resistance in EOC cells, can STAT3 affect the chemotherapy resistance of EOC cells by regulating other miRNAs?

## Data Availability

Not applicable.

## Abbreviations

STAT3: Signal transducer and activator of transcription 3
NcRNA: Noncoding RNA
LncRNA: Long noncoding RNA
miRNA: MicroRNA
circRNA: Circular RNA
NSCLC: Non-small cell lung cancer
CRC: Colorectal cancer
ceRNA: Competitive endogenous RNA
KLF7: Encoding KLF transcription factor 7
BC: Breast cancer
EGFR: Encoding epidermal growth factor receptor
TSS: Transcription start site
OGT: *O*-GlcNAcy transfer
VSMCs: Vascular smooth muscle cells
AAA: Abdominal aortic aneurysm
HUVECs: Human umbilical vein endothelial cells
AS: Atherosclerosis
PKC-α: Protein kinase C α
IL17A: Encoding interleukin 17A
IL17F: Encoding interleukin 17F
CCR6: Encoding C–C motif chemokine receptor 6
CXCL13: Encoding C-X-C motif chemokine ligand 13
RA: Rheumatoid arthritis
PKA: Protein kinase A
CREB: CAMP response element binding protein
EMT: Epithelial–mesenchymal transition
CSCC: Cervical squamous cell carcinoma
UTRs: Untranslated regions
TGFBR3: Encoding transforming growth factor beta receptor 3
HMGCS1: Encoding 3-hydroxy-3-methylglutaryl-CoA synthase 1
CAC: Colorectal cancer
TET3: Encoding Tet methylcytosine dioxygenase 3
5hmC: 5-Hydroxymethylcytosine
HCC: Hepatocellular carcinoma
PGC1A: Encoding PPARG coactivator 1 alpha
G6PC: Encoding glucose-6-phosphatase
HRECs: Human retinal endothelial cells
TIMP3: Encoding tissue inhibitor of matrix metalloproteinase-3
IL6R: Encoding IL-6 receptor
EOC: Epithelial ovarian cancer
EZH2: Enhancer of zeste 2 polycomb repressive complex 2 subunit
OV: Ovarian cancer
GC: Gastric cancer
RCC: Renal cell carcinoma
GATA3: GATA binding protein 3
GLAST: Glutamate/aspartate transporter 1
ITP: Immune thrombocytopenia
ECS: Extracapsular nodal spread
ALL: Acute lymphoblastic leukemia
SOCS3: Suppressor of cytokine signaling 3
PIAS3: Protein inhibitor of activated STAT3
JAK2: Janus kinase 2
RBPs: RNA binding proteins
SCC: Squamous cell carcinoma
SHP-1: Src homology 2 domain-containing protein tyrosine phosphatase 1

## References

[1] de Los G, Fayos Alonso I, Zujo L, Wiest I, Kodajova P, Timelthaler G, Edtmayer S, Zrimsek M, Kollmann S, Giordano C, Kothmayer M (2022). PDGFRbeta promotes oncogenic progression via STAT3/STAT5 hyperactivation in anaplastic large cell lymphoma. Mol Cancer.

[2] Dawson RE, Deswaerte V, West AC, Tang K, West AJ, Balic JJ, Gearing LJ, Saad MI, Yu L, Wu Y (2022). STAT3-mediated upregulation of the AIM2 DNA sensor links innate immunity with cell migration to promote epithelial tumourigenesis. Gut.

[3] Qureshy Z, Li H, Zeng Y, Rivera J, Cheng N, Peterson CN, Kim MO, Ryan WR, Ha PK, Bauman JE (2022). STAT3 activation as a predictive biomarker for ruxolitinib response in head and neck cancer. Clin Cancer Res.

[4] Liu Y, Xu Q, Deng F, Zheng Z, Luo J, Wang P, Zhou J, Lu X, Zhang L, Chen Z (2023). HERC2 promotes inflammation-driven cancer stemness and immune evasion in hepatocellular carcinoma by activating STAT3 pathway. J Exp Clin Cancer Res.

[5] Slack FJ, Chinnaiyan AM (2019). The role of non-coding RNAs in oncology. Cell.

[6] Poller W, Dimmeler S, Heymans S, Zeller T, Haas J, Karakas M, Leistner DM, Jakob P, Nakagawa S, Blankenberg S (2018). Non-coding RNAs in cardiovascular diseases: diagnostic and therapeutic perspectives. Eur Heart J.

[7] Costales MG, Hoch DG, Abegg D, Childs-Disney JL, Velagapudi SP, Adibekian A, Disney MD (2019). A designed small molecule inhibitor of a non-coding RNA sensitizes HER2 negative cancers to herceptin. J Am Chem Soc.

[8] Tan YT, Lin JF, Li T, Li JJ, Xu RH, Ju HQ (2020). LncRNA-mediated posttranslational modifications and reprogramming of energy metabolism in cancer. Cancer Commun.

[9] Adams BD, Wali VB, Cheng CJ, Inukai S, Booth CJ, Agarwal S, Rimm DL, Gyorffy B, Santarpia L, Pusztai L (2016). miR-34a silences c-SRC to attenuate tumor growth in triple-negative breast cancer. Cancer Res.

[10] Hill M, Tran N (2021). Global miRNA to miRNA interactions: impacts for miR-21. Trends Cell Biol.

[11] Chen B, Deng S, Ge T, Ye M, Yu J, Lin S, Ma W, Songyang Z (2020). Live cell imaging and proteomic profiling of endogenous NEAT1 lncRNA by CRISPR/Cas9-mediated knock-in. Protein Cell.

[12] Anandappa G, Lampis A, Cunningham D, Khan KH, Kouvelakis K, Vlachogiannis G, Hedayat S, Tunariu N, Rao S, Watkins D (2019). miR-31-3p expression and benefit from anti-EGFR inhibitors in metastatic colorectal cancer patients enrolled in the prospective phase II PROSPECT-C trial. Clin Cancer Res.

[13] Xu X, Zhang J, Tian Y, Gao Y, Dong X, Chen W, Yuan X, Yin W, Xu J, Chen K (2020). CircRNA inhibits DNA damage repair by interacting with host gene. Mol Cancer.

[14] Zhang MW, Zhu ZH, Xia ZK, Yang X, Luo WT, Ao JH, Yang RY (2021). Comprehensive circRNA-microRNA-mRNA network analysis revealed the novel regulatory mechanism of *Trichosporon asahii* infection. Mil Med Res.

[15] Lei M, Zheng G, Ning Q, Zheng J, Dong D (2020). Translation and functional roles of circular RNAs in human cancer. Mol Cancer.

[16] Kleaveland B, Shi CY, Stefano J, Bartel DP (2018). A network of noncoding regulatory RNAs acts in the mammalian brain. Cell.

[17] Wang Q, Liu J, You Z, Yin Y, Liu L, Kang Y, Li S, Ning S, Li H, Gong Y (2021). LncRNA TINCR favors tumorigenesis via STAT3-TINCR-EGFR-feedback loop by recruiting DNMT1 and acting as a competing endogenous RNA in human breast cancer. Cell Death Dis.

[18] An YX, Shang YJ, Xu ZW, Zhang QC, Wang Z, Xuan WX, Zhang XJ (2019). STAT3-induced long noncoding RNA LINC00668 promotes migration and invasion of non-small cell lung cancer via the miR-193a/KLF7 axis. Biomed Pharmacother.

[19] Zheng W, Li H, Zhang H, Zhang C, Zhu Z, Liang H, Zhou Y (2020). Long noncoding RNA RHPN1-AS1 promotes colorectal cancer progression via targeting miR-7-5p/OGT axis. Cancer Cell Int.

[20] Yang B, Wang X, Ying C, Peng F, Xu M, Chen F, Cai B (2021). Long noncoding RNA SNHG16 facilitates abdominal aortic aneurysm progression through the miR-106b-5p/STAT3 feedback loop. J Atheroscler Thromb.

[21] Khan MM, Khan MH, Kalim UU, Khan S, Junttila S, Paulin N, Kong L, Rasool O, Elo LL, Lahesmaa R (2022). Long intergenic noncoding RNA MIAT as a regulator of human Th17 cell differentiation. Front Immunol.

[22] Chen Y, Li H, Ding T, Li J, Zhang Y, Wang J, Yang X, Chong T, Long Y, Li X (2020). Lnc-M2 controls M2 macrophage differentiation via the PKA/CREB pathway. Mol Immunol.

[23] Putoczki Tracy L, Thiem S, Loving A, Busuttil Rita A, Wilson Nicholas J, Ziegler Paul K, Nguyen PM, Preaudet A, Farid R, Edwards KM (2013). Interleukin-11 Is the dominant IL-6 family cytokine during gastrointestinal tumorigenesis and can be targeted therapeutically. Cancer Cell.

[24] Yu H, Lee H, Herrmann A, Buettner R, Jove R (2014). Revisiting STAT3 signalling in cancer: new and unexpected biological functions. Nat Rev Cancer.

[25] Ma J-h, Qin L, Li X (2020). Role of STAT3 signaling pathway in breast cancer. Cell Commun Signal.

[26] Xie C, Zhou X, Liang C, Li X, Ge M, Chen Y, Yin J, Zhu J, Zhong C (2021). Apatinib triggers autophagic and apoptotic cell death via VEGFR2/STAT3/PD-L1 and ROS/Nrf2/p62 signaling in lung cancer. J Exp Clin Cancer Res.

[27] Zhong Q, Fang Y, Lai Q, Wang S, He C, Li A, Liu S, Yan Q (2020). CPEB3 inhibits epithelial-mesenchymal transition by disrupting the crosstalk between colorectal cancer cells and tumor-associated macrophages via IL-6R/STAT3 signaling. J Exp Clin Cancer Res.

[28] Wei C, Yang C, Wang S, Shi D, Zhang C, Lin X, Liu Q, Dou R, Xiong B (2019). Crosstalk between cancer cells and tumor associated macrophages is required for mesenchymal circulating tumor cell-mediated colorectal cancer metastasis. Mol Cancer.

[29] Li B, Huang N, Wei S, Xv J, Meng Q, Aschner M, Li X, Chen R (2021). lncRNA TUG1 as a ceRNA promotes PM exposure-induced airway hyper-reactivity. J Hazard Mater.

[30] Alvarado FJ, Valdivia CR, Valdivia HH (2018). Navigating the sea of long noncoding RNAs: ZFAS1, friend or foe?. Circ Res.

[31] Liu H, Li C, Yang J, Sun Y, Zhang S, Yang J, Yang L, Wang Y, Jiao B (2018). Long noncoding RNA CASC9/miR-519d/STAT3 positive feedback loop facilitate the glioma tumourigenesis. J Cell Mol Med.

[32] Xie Q, Li F, Shen K, Luo C, Song G (2020). LOXL1-AS1/miR-515-5p/STAT3 positive feedback loop facilitates cell proliferation and migration in atherosclerosis. J Cardiovasc Pharmacol.

[33] Hanlon MM, Rakovich T, Cunningham CC, Ansboro S, Veale DJ, Fearon U, McGarry T (2019). STAT3 mediates the differential effects of oncostatin M and TNFalpha on RA synovial fibroblast and endothelial cell function. Front Immunol.

[34] Damasceno LEA, Prado DS, Veras FP, Fonseca MM, Toller-Kawahisa JE, Rosa MH, Publio GA, Martins TV, Ramalho FS, Waisman A (2020). PKM2 promotes Th17 cell differentiation and autoimmune inflammation by fine-tuning STAT3 activation. J Exp Med.

[35] Meisel M, Hermann-Kleiter N, Hinterleitner R, Gruber T, Wachowicz K, Pfeifhofer-Obermair C, Fresser F, Leitges M, Soldani C, Viola A (2013). The kinase PKCα selectively upregulates interleukin-17A during Th17 cell immune responses. Immunity.

[36] Zhang J, Jiang M, Qian L, Lin X, Song W, Gao Y, Zhou Y (2020). The STAT3-miR-223-TGFBR3/HMGCS1 axis modulates the progression of cervical carcinoma. Mol Oncol.

[37] Wang A, Deng S, Chen X, Yu C, Du Q, Wu Y, Chen G, Hu L, Hu C, Li Y (2020). miR-29a-5p/STAT3 positive feedback loop regulates TETs in colitis-associated colorectal cancer. Inflamm Bowel Dis.

[38] Wang B, Hsu SH, Frankel W, Ghoshal K, Jacob ST (2012). Stat3-mediated activation of microRNA-23a suppresses gluconeogenesis in hepatocellular carcinoma by down-regulating glucose-6-phosphatase and peroxisome proliferator-activated receptor gamma, coactivator 1 alpha. Hepatology.

[39] Gutsaeva DR, Thounaojam M, Rajpurohit S, Powell FL, Martin PM, Goei S, Duncan M, Bartoli M (2017). STAT3-mediated activation of miR-21 is involved in down-regulation of TIMP3 and neovascularization in the ischemic retina. Oncotarget.

[40] Rokavec M, Oner MG, Li H, Jackstadt R, Jiang L, Lodygin D, Kaller M, Horst D, Ziegler PK, Schwitalla S (2014). IL-6R/STAT3/miR-34a feedback loop promotes EMT-mediated colorectal cancer invasion and metastasis. J Clin Invest.

[41] Zhu X, Shen H, Yin X, Long L, Chen X, Feng F, Liu Y, Zhao P, Xu Y, Li M (2017). IL-6R/STAT3/miR-204 feedback loop contributes to cisplatin resistance of epithelial ovarian cancer cells. Oncotarget.

[42] Sabatel C, Malvaux L, Bovy N, Deroanne C, Lambert V, Gonzalez ML, Colige A, Rakic JM, Noel A, Martial JA, Struman I (2011). MicroRNA-21 exhibits antiangiogenic function by targeting RhoB expression in endothelial cells. PLoS ONE.

[43] Achkar IW, Abdulrahman N, Al-Sulaiti H, Joseph JM, Uddin S, Mraiche F (2018). Cisplatin based therapy: the role of the mitogen activated protein kinase signaling pathway. J Transl Med.

[44] Sun C-Y, Nie J, Huang J-P, Zheng G-J, Feng B (2019). Targeting STAT3 inhibition to reverse cisplatin resistance. Biomed Pharmacother.

[45] Bosch-Barrera J, Queralt B, Menendez JA (2017). Targeting STAT3 with silibinin to improve cancer therapeutics. Cancer Treat Rev.

[46] Kim E, Kim M, Woo D-H, Shin Y, Shin J, Chang N, Oh Young T, Kim H, Rheey J, Nakano I (2013). Phosphorylation of EZH2 activates STAT3 signaling via STAT3 methylation and promotes tumorigenicity of glioblastoma stem-like cells. Cancer Cell.

[47] Sun S, Gao J, Zhou S, Li Y, Wang Y, Jin L, Li J, Liu B, Zhang B, Han S (2020). A novel circular RNA circ-LRIG3 facilitates the malignant progression of hepatocellular carcinoma by modulating the EZH2/STAT3 signaling. J Exp Clin Cancer Res.

[48] Wang Y, Zhao W, Zhang S (2020). STAT3-induced upregulation of circCCDC66 facilitates the progression of non-small cell lung cancer by targeting miR-33a-5p/KPNA4 axis. Biomed Pharmacother.

[49] Zhang Y, Cheng X, Liang H, Jin Z (2018). Long non-coding RNA HOTAIR and STAT3 synergistically regulate the cervical cancer cell migration and invasion. Chem Biol Interact.

[50] Liu Z, Li H, Fan S, Lin H, Lian W (2019). STAT3-induced upregulation of long noncoding RNA HNF1A-AS1 promotes the progression of oral squamous cell carcinoma via activating Notch signaling pathway. Cancer Biol Ther.

[51] Lee JH, Kim JE, Kim BG, Han HH, Kang S, Cho NH (2016). STAT3-induced WDR1 overexpression promotes breast cancer cell migration. Cell Signal.

[52] Wu K, Zhang N, Ma J, Huang J, Chen J, Wang L, Zhang J (2018). Long noncoding RNA FAL1 promotes proliferation and inhibits apoptosis of human colon cancer cells. IUBMB Life.

[53] Zhao X, Cheng Z, Wang J (2018). Long noncoding RNA FEZF1-AS1 promotes proliferation and inhibits apoptosis in ovarian cancer by activation of JAK-STAT3 pathway. Med Sci Monit.

[54] Sun D, Liu H, Wang T (2020). Long noncoding RNA RP11-334E6.12 promotes the proliferation, migration and invasion of breast cancer cells through the EMT pathway by activating the STAT3 cascade. Cancer Manag Res.

[55] Liu C, Lin P, Zhao J, Xie H, Li R, Yang X, Wang N, Jia H, Jiang S, Zhang K, Yu X (2021). Knockdown of long noncoding RNA AC245100.4 inhibits the tumorigenesis of prostate cancer cells via the STAT3/NR4A3 axis. Epigenomics.

[56] Luo Z, Cao P (2019). Long noncoding RNA PVT1 promotes hepatoblastoma cell proliferation through activating STAT3. Cancer Manag Res.

[57] Wu K, Xu K, Liu K, Huang J, Chen J, Zhang J, Zhang N (2018). Long noncoding RNA BC200 regulates cell growth and invasion in colon cancer. Int J Biochem Cell Biol.

[58] Zhao J, Wu J, Qin Y, Zhang W, Huang G, Qin L (2020). LncRNA PVT1 induces aggressive vasculogenic mimicry formation through activating the STAT3/Slug axis and epithelial-to-mesenchymal transition in gastric cancer. Cell Oncol (Dordr).

[59] Jin S, Yang X, Li J, Yang W, Ma H, Zhang Z (2019). p53-targeted lincRNA-p21 acts as a tumor suppressor by inhibiting JAK2/STAT3 signaling pathways in head and neck squamous cell carcinoma. Mol Cancer.

[60] Li JP, Xiang Y, Fan LJ, Yao A, Li H, Liao XH (2019). Long noncoding RNA H19 competitively binds miR-93-5p to regulate STAT3 expression in breast cancer. J Cell Biochem.

[61] Hua K, Deng X, Hu J, Ji C, Yu Y, Li J, Wang X, Fang L (2020). Long noncoding RNA HOST2, working as a competitive endogenous RNA, promotes STAT3-mediated cell proliferation and migration via decoying of let-7b in triple-negative breast cancer. J Exp Clin Cancer Res.

[62] Zhang XN, Zhou J, Lu XJ (2018). The long noncoding RNA NEAT1 contributes to hepatocellular carcinoma development by sponging miR-485 and enhancing the expression of the STAT3. J Cell Physiol.

[63] Lin Q, Zheng H, Xu J, Zhang F, Pan H (2019). LncRNA SNHG16 aggravates tumorigenesis and development of hepatocellular carcinoma by sponging miR-4500 and targeting STAT3. J Cell Biochem.

[64] Zhang X, Pan B, Qiu J, Ke X, Shen S, Wang X, Tang N (2022). lncRNA MIAT targets miR-411-5p/STAT3/PD-L1 axis mediating hepatocellular carcinoma immune response. Int J Exp Pathol.

[65] Tan HY, Wang C, Liu G, Zhou X (2019). Long noncoding RNA NEAT1-modulated miR-506 regulates gastric cancer development through targeting STAT3. J Cell Biochem.

[66] Tian P, Wei JX, Li J, Ren JK, Yang JJ (2021). LncRNA SNHG1 regulates immune escape of renal cell carcinoma by targeting miR-129-3p to activate STAT3 and PD-L1. Cell Biol Int.

[67] Zhou W, Wang L, Miao Y, Xing R (2018). Novel long noncoding RNA GACAT3 promotes colorectal cancer cell proliferation, invasion, and migration through miR-149. Onco Targets Ther.

[68] Liu Y, Chen L, Yuan H, Guo S, Wu G (2020). LncRNA DANCR promotes sorafenib resistance via activation of IL-6/STAT3 signaling in hepatocellular carcinoma cells. Onco Targets Ther.

[69] Yang L, Gao Q, Wu X, Feng F, Xu K (2018). Long noncoding RNA HEGBC promotes tumorigenesis and metastasis of gallbladder cancer via forming a positive feedback loop with IL-11/STAT3 signaling pathway. J Exp Clin Cancer Res.

[70] Wu J, Zhang J, Shen B, Yin K, Xu J, Gao W, Zhang L (2015). Long noncoding RNA lncTCF7, induced by IL-6/STAT3 transactivation, promotes hepatocellular carcinoma aggressiveness through epithelial-mesenchymal transition. J Exp Clin Cancer Res.

[71] DeVaux RS, Ropri AS, Grimm SL, Hall PA, Herrera EO, Chittur SV, Smith WP, Coarfa C, Behbod F, Herschkowitz JI (2020). Long noncoding RNA BHLHE40-AS1 promotes early breast cancer progression through modulating IL-6/STAT3 signaling. J Cell Biochem.

[72] Wang J, Zhou J, Jiang C, Zheng J, Namba H, Chi P, Asakawa T (2019). LNRRIL6, a novel long noncoding RNA, protects colorectal cancer cells by activating the IL-6-STAT3 pathway. Mol Oncol.

[73] Li MX, Wang HY, Yuan CH, Ma ZL, Jiang B, Li L, Zhang L, Xiu DR (2021). KLHDC7B-DT aggravates pancreatic ductal adenocarcinoma development via inducing cross-talk between cancer cells and macrophages. Clin Sci (Lond).

[74] Wang S, Liang K, Hu Q, Li P, Song J, Yang Y, Yao J, Mangala LS, Li C, Yang W (2017). JAK2-binding long noncoding RNA promotes breast cancer brain metastasis. J Clin Invest.

[75] Xu Z, Yang F, Wei D, Liu B, Chen C, Bao Y, Wu Z, Wu D, Tan H, Li J (2017). Long noncoding RNA-SRLR elicits intrinsic sorafenib resistance via evoking IL-6/STAT3 axis in renal cell carcinoma. Oncogene.

[76] Yuan JH, Yang F, Wang F, Ma JZ, Guo YJ, Tao QF, Liu F, Pan W, Wang TT, Zhou CC (2014). A long noncoding RNA activated by TGF-beta promotes the invasion-metastasis cascade in hepatocellular carcinoma. Cancer Cell.

[77] Li Y, Ye Y, Chen H (2018). Astragaloside IV inhibits cell migration and viability of hepatocellular carcinoma cells via suppressing long noncoding RNA ATB. Biomed Pharmacother.

[78] Chen T, Pei J, Wang J, Luo R, Liu L, Wang L, Jia H (2019). HBx-related long non-coding RNA 01152 promotes cell proliferation and survival by IL-23 in hepatocellular carcinoma. Biomed Pharmacother.

[79] Zhang J, Li Z, Liu L, Wang Q, Li S, Chen D, Hu Z, Yu T, Ding J, Li J (2018). Long noncoding RNA TSLNC8 is a tumor suppressor that inactivates the interleukin-6/STAT3 signaling pathway. Hepatology.

[80] Lin D, Zhang H, Zhang J, Huang K, Chen Y, Jing X, Tao E (2023). alpha-Synuclein induces neuroinflammation injury through the IL6ST-AS/STAT3/HIF-1alpha axis. Int J Mol Sci.

[81] Shui X, Chen S, Lin J, Kong J, Zhou C, Wu J (2019). Knockdown of lncRNA NEAT1 inhibits Th17/CD4(+) T cell differentiation through reducing the STAT3 protein level. J Cell Physiol.

[82] Li J, Tian J, Lu J, Wang Z, Ling J, Wu X, Yang F, Xia Y (2020). LncRNA GAS5 inhibits Th17 differentiation and alleviates immune thrombocytopenia via promoting the ubiquitination of STAT3. Int Immunopharmacol.

[83] Zhang W, Zheng X, Yu Y, Zheng L, Lan J, Wu Y, Liu H, Zhao A, Huang H, Chen W (2022). Renal cell carcinoma-derived exosomes deliver lncARSR to induce macrophage polarization and promote tumor progression via STAT3 pathway. Int J Biol Sci.

[84] Liu B, Liu Q, Pan S, Huang Y, Qi Y, Li S, Xiao Y, Jia L (2019). The HOTAIR/miR-214/ST6GAL1 crosstalk modulates colorectal cancer procession through mediating sialylated c-Met via JAK2/STAT3 cascade. J Exp Clin Cancer Res.

[85] Shi C, Zhao Y, Li Q, Li J (2021). lncRNA SNHG14 Plays a role in sepsis-induced acute kidney injury by regulating miR-93. Mediators Inflamm.

[86] Li JL, Liu XL, Guo SF, Yang Y, Zhu YL, Li JZ (2019). Long noncoding RNA UCA1 regulates proliferation and apoptosis in multiple myeloma by targeting miR-331–3p/IL6R axis for the activation of JAK2/STAT3 pathway. Eur Rev Med Pharmacol Sci.

[87] Huang H, Zhang G, Ge Z (2021). lncRNA MALAT1 promotes renal fibrosis in diabetic nephropathy by targeting the miR-2355-3p/IL6ST axis. Front Pharmacol.

[88] Lu P, Gu Y, Li L, Wang F, Yang X, Yang Y (2018). Long noncoding RNA CAMTA1 promotes proliferation and mobility of the human breast cancer cell line MDA-MB-231 via targeting miR-20b. Oncol Res.

[89] Sun H, Shi K, Xie D, Zhang H, Yu B (2019). Long noncoding RNA C2dat1 protects H9c2 cells against hypoxia injury by downregulating miR-22. J Cell Physiol.

[90] Ke S, Fang M, Li R, Wang J, Lu J (2022). Downregulation of long noncoding RNA breast cancer anti-estrogen resistance 4 inhibits cell proliferation, invasion, and migration in esophageal squamous cell carcinoma by regulating the microRNA-181c-5p/LIM and SH3 protein 1 axis. Bioengineered.

[91] Jia B, Dao J, Han J, Huang Z, Sun X, Zheng X, Xiang S, Zhou H, Liu S (2021). LINC00958 promotes the proliferation of TSCC via miR-211-5p/CENPK axis and activating the JAK/STAT3 signaling pathway. Cancer Cell Int.

[92] Zhang XW, Wang L, Ding H (2019). Long noncoding RNA AK089579 inhibits epithelial-to-mesenchymal transition of peritoneal mesothelial cells by competitively binding to microRNA-296-3p via DOK2 in peritoneal fibrosis. FASEB J.

[93] Zhang S, Ji WW, Wei W, Zhan LX, Huang X (2021). Long noncoding RNA Meg3 sponges miR-708 to inhibit intestinal tumorigenesis via SOCS3-repressed cancer stem cells growth. Cell Death Dis.

[94] Huang G, Wu X, Li S, Xu X, Zhu H, Chen X (2016). The long noncoding RNA CASC2 functions as a competing endogenous RNA by sponging miR-18a in colorectal cancer. Sci Rep.

[95] Chi F, Qiu F, Jin X, Chen L, He G, Han S (2022). LINC00982 inhibits the proliferation, migration, and invasion of breast cancer cells through the miR-765/DPF3 axis. DNA Cell Biol.

[96] Dai R, Jiang Q, Zhou Y, Lin R, Lin H, Zhang Y, Zhang J, Gao X (2021). Lnc-STYK1-2 regulates bladder cancer cell proliferation, migration, and invasion by targeting miR-146b-5p expression and AKT/STAT3/NF-kB signaling. Cancer Cell Int.

[97] Si J, Ma Y, Lv C, Hong Y, Tan H, Yang Y (2021). HIF1A-AS2 induces osimertinib resistance in lung adenocarcinoma patients by regulating the miR-146b-5p/IL-6/STAT3 axis. Mol Ther Nucleic Acids.

[98] Zheng R, Jia J, Guan L, Yuan H, Liu K, Liu C, Ye W, Liao Y, Lin S, Huang O (2020). Long noncoding RNA lnc-LOC645166 promotes adriamycin resistance via NF-kappaB/GATA3 axis in breast cancer. Aging (Albany NY).

[99] Zhang L, Ye F, Zuo Z, Cao D, Peng Y, Li Z, Huang J, Duan L (2021). Long noncoding RNA TPT1-AS1 promotes the progression and metastasis of colorectal cancer by upregulating the TPT1-mediated FAK and JAK-STAT3 signalling pathways. Aging (Albany NY)..

[100] Li R, Chen S, Zhan J, Li X, Liu W, Sheng X, Lu Z, Zhong R, Chen L, Luo X (2020). Long noncoding RNA FOXD2-AS1 enhances chemotherapeutic resistance of laryngeal squamous cell carcinoma via STAT3 activation. Cell Death Dis.

[101] Zhang X, Jiang Y, Xie Y, Leng X, He M, Song F (2021). Inhibition of gastric cancer cell apoptosis by long noncoding RNA TRPM2-AS via mitogen-activated protein kinase and activators of transduction-3. J Gastroenterol Hepatol.

[102] Zhuang L, Tian J, Zhang X, Wang H, Huang C (2018). Lnc-DC regulates cellular turnover and the HBV-induced immune response by TLR9/STAT3 signaling in dendritic cells. Cell Mol Biol Lett.

[103] Piipponen M, Nissinen L, Riihila P, Farshchian M, Kallajoki M, Peltonen J, Peltonen S, Kahari VM (2020). p53-regulated long noncoding RNA PRECSIT promotes progression of cutaneous squamous cell carcinoma via STAT3 signaling. Am J Pathol.

[104] Wang H, Yao G, Li L, Ma Z, Chen J, Chen W (2020). LncRNA-UCA1 inhibits the astrocyte activation in the temporal lobe epilepsy via regulating the JAK/STAT signaling pathway. J Cell Biochem.

[105] Su K, Zhao Q, Bian A, Wang C, Cai Y, Zhang Y (2018). A novel positive feedback regulation between long noncoding RNA UICC and IL-6/STAT3 signaling promotes cervical cancer progression. Am J Cancer Res.

[106] Csermely P, Wei J, Yuan Y, Jin C, Chen H, Leng L, He F, Wang J (2012). The ubiquitin ligase TRAF6 negatively regulates the JAK-STAT signaling pathway by binding to STAT3 and mediating its ubiquitination. PLoS ONE.

[107] Dong P, Xiong Y, Yue J, Xu D, Ihira K, Konno Y, Kobayashi N, Todo Y, Watari H (2019). Long noncoding RNA NEAT1 drives aggressive endometrial cancer progression via miR-361-regulated networks involving STAT3 and tumor microenvironment-related genes. J Exp Clin Cancer Res.

[108] Zhao D, Xing Q, Song H, Zhao Y, Guo G (2021). LINC00265/miR-4500 axis accelerates acute lymphoblastic leukemia progression by enhancing STAT3 signals. Cancer Manag Res.

[109] Wu Y, Xu J, Xu J, Cheng J, Jiao D, Zhou C, Dai Y, Chen Q (2017). Lower serum levels of miR-29c-3p and miR-19b-3p as biomarkers for Alzheimer’s disease. Tohoku J Exp Med.

[110] Yang Q, Zhao S, Shi Z, Cao L, Liu J, Pan T, Zhou D, Zhang J (2021). Chemotherapy-elicited exosomal miR-378a-3p and miR-378d promote breast cancer stemness and chemoresistance via the activation of EZH2/STAT3 signaling. J Exp Clin Cancer Res.

[111] Yang Y, Ding L, Hu Q, Xia J, Sun J, Wang X, Xiong H, Gurbani D, Li L, Liu Y, Liu A (2017). MicroRNA-218 functions as a tumor suppressor in lung cancer by targeting IL-6/STAT3 and negatively correlates with poor prognosis. Mol Cancer.

[112] Wang F, Rong L, Zhang Z, Li M, Ma L, Ma Y, Xie X, Tian X, Yang Y (2020). LncRNA H19-derived miR-675-3p promotes epithelial-mesenchymal transition and stemness in human pancreatic cancer cells by targeting the STAT3 pathway. J Cancer.

[113] Collins AS, McCoy CE, Lloyd AT, O'Farrelly C, Stevenson NJ (2013). miR-19a: an effective regulator of SOCS3 and enhancer of JAK-STAT signalling. PLoS ONE.

[114] Zhang J, Jia J, Zhao L, Li X, Xie Q, Chen X, Wang J, Lu F (2016). Down-regulation of microRNA-9 leads to activation of IL-6/Jak/STAT3 pathway through directly targeting IL-6 in HeLa cell. Mol Carcinog.

[115] Zhang JF, He ML, Fu WM, Wang H, Chen LZ, Zhu X, Chen Y, Xie D, Lai P, Chen G (2011). Primate-specific microRNA-637 inhibits tumorigenesis in hepatocellular carcinoma by disrupting signal transducer and activator of transcription 3 signaling. Hepatology.

[116] Zhang X, Sai B, Wang F, Wang L, Wang Y, Zheng L, Li G, Tang J, Xiang J (2019). Hypoxic BMSC-derived exosomal miRNAs promote metastasis of lung cancer cells via STAT3-induced EMT. Mol Cancer.

[117] Tian K, Liu W, Zhang J, Fan X, Liu J, Zhao N, Yao C, Miao G (2020). MicroRNA-125b exerts antitumor functions in cutaneous squamous cell carcinoma by targeting the STAT3 pathway. Cell Mol Biol Lett.

[118] Suwei D, Yanbin X, Jianqiang W, Xiang M, Zhuohui P, Jianping K, Yunqing W, Zhen L (2022). Metformin inhibits melanoma cell metastasis by suppressing the miR-5100/SPINK5/STAT3 axis. Cell Mol Biol Lett.

[119] Galoczova M, Coates P, Vojtesek B (2018). STAT3, stem cells, cancer stem cells and p63. Cell Mol Biol Lett.

[120] Cai B, Li J, Wang J, Luo X, Ai J, Liu Y, Wang N, Liang H, Zhang M, Chen N (2012). microRNA-124 regulates cardiomyocyte differentiation of bone marrow-derived mesenchymal stem cells via targeting STAT3 signaling. Stem Cells.

[121] Zhang H, Cai K, Wang J, Wang X, Cheng K, Shi F, Jiang L, Zhang Y, Dou J (2014). MiR-7, inhibited indirectly by LincRNA HOTAIR, directly inhibits SETDB1 and reverses the EMT of breast cancer stem cells by downregulating the STAT3 pathway. Stem Cells.

[122] Jiang J, Li Z, Yu C, Chen M, Tian S, Sun C (2015). MiR-1181 inhibits stem cell-like phenotypes and suppresses SOX2 and STAT3 in human pancreatic cancer. Cancer Lett.

[123] Zhang Y, Qian L, Liu Y, Liu Y, Yu W, Zhao Y (2021). CircRNA-ceRNA network revealing the potential regulatory roles of CircRNA in Alzheimer's disease involved the cGMP-PKG signal pathway. Front Mol Neurosci.

[124] Xu Y, Jiang T, Wu C, Zhang Y (2020). CircAKT3 inhibits glycolysis balance in lung cancer cells by regulating miR-516b-5p/STAT3 to inhibit cisplatin sensitivity. Biotechnol Lett.

[125] Zhang H, Ge Z, Wang Z, Gao Y, Wang Y, Qu X (2021). Circular RNA RHOT1 promotes progression and inhibits ferroptosis via mir-106a-5p/STAT3 axis in breast cancer. Aging (Albany NY).

[126] Wang J, Zhang Y, Song H, Yin H, Jiang T, Xu Y, Liu L, Wang H, Gao H, Wang R, Song J (2021). The circular RNA circSPARC enhances the migration and proliferation of colorectal cancer by regulating the JAK/STAT pathway. Mol Cancer.

[127] Yang J, Zhang X, Cao J, Xu P, Chen Z, Wang S, Li B, Zhang L, Xie L, Fang L, Xu Z (2021). Circular RNA UBE2Q2 promotes malignant progression of gastric cancer by regulating signal transducer and activator of transcription 3-mediated autophagy and glycolysis. Cell Death Dis.

[128] Chen X, Mao R, Su W, Yang X, Geng Q, Guo C, Wang Z, Wang J, Kresty LA, Beer DG (2020). Circular RNA circHIPK3 modulates autophagy via MIR124-3p-STAT3-PRKAA/AMPKalpha signaling in STK11 mutant lung cancer. Autophagy.

[129] Zhou X-Y, Yang H, Bai Y-Q, Li X-L, Han S-Y, Zhou B-X (2020). hsa_circ_0006916 promotes hepatocellular carcinoma progression by activating the miR-337–3p/STAT3 axis. Cell Mol Biol Lett.

[130] Hu C, Xia R, Zhang X, Li T, Ye Y, Li G, He R, Li Z, Lin Q, Zheng S, Chen R (2022). circFARP1 enables cancer-associated fibroblasts to promote gemcitabine resistance in pancreatic cancer via the LIF/STAT3 axis. Mol Cancer.

[131] Jia L, Wang Y, Wang CY (2021). circFAT1 promotes cancer stemness and immune evasion by promoting STAT3 activation. Adv Sci (Weinh).

